# The Cholera as It Appeared at the Port of New York in 1865

**Published:** 1866-03

**Authors:** J. Swinburne

**Affiliations:** Port Physician


*The Cholera as it appeared at the Port of New York in* 1865.
By J. SWINBURNE, M. D., Port Physician.

The "Atlanta," an English mail steamer, iron-built, of 325 feet in length, and 36 feet beam, with two first and second cabins fore and aft on the deck, and three separate steerages of 98, 80 and 70 feet in length, and 8½ to 9 feet in height, sailed from London on the 10th of October, with a full cargo, and 28 cabin and 12 steerage passengers.

London was at that time perfectly healthy.

On the 11th she arrived at Havre, remaining only one day, and receiving 24 additional cabin and 540 steerage passengers, mostly from Switzerland, the southern part of Germany and eastern France, all, with few exceptions, passing through Paris on their way to Havre, some remaining only a few hours, others for days in the metropolis, when already at that time the cholera was reported to prevail, though to a limited extent, and of a mild type. Among these were two families from Germany who remained a day at the hotel "City of New York," at Paris, and five days at the "Weissen Lamm" and "Hullgarder Hof," in Havre. While at these hotels emigrants who had arrived only a few days before them were taken ill, visited and attended by government officials, and by their orders sent to the hospitals.

The "Atlanta" sailed again on the 12th of October.

On the 13th the first death from cholera occurred in the person of a little child in the family from the "Weissen Lamm."

On the 14th, 16th, 18th, 19th and 22d, five deaths from cholera occurred in one family from the "Hullgarder Hof."

On the 22d a friend of the family, also from the "Hullgarder Hof," but in the 2d steerage, sickened, and died on the 24th.

On the 28th the first case occurred in the 3d steerage; 3 of the emigrants from London were taken ill on the 30th, all of whom, however, recovered.

When the "Atlanta" arrived the Surgeon reported 60 cases of cholera and 15 deaths during the passage; two more died after her arrival in port, and 6 out of 42 cases admitted on board the hospital ships, making a total of 102 cases and 23 deaths. Of the 42 cases treated in the hospital 22 were admitted on the 6th; six on the 7th; two on the 8th; seven on the 9th; two on the 15th; three on the 16th; one on the 19th.

From the first case the disease presented the uniform symptoms pathognostic of Asiatic cholera, and although in comparatively few cases terminating fatally, the same virus produced the milder forms of disease, which destroyed life in twenty-four, or even twelve hours.

The "Hermann," which sailed from Havre at the same time with the "Atlanta," arrived at the lower quarantine on the 26th of November. The physician in charge reported 7 deaths—4 children and 3 adults. The former he reported to have died of diarrhœa and inanition; the 3 adults of disease of the heart, inflammation of the bowels, and premature parturition after a few days illness. Singular, however, that the first death occurred in the very family who had lost the mother at the Hullgarder Hof at Havre, and whose disease and death, after thirty-six hours' illness, the illiterate peasant, her husband, so graphically described, that no doubt whatever could exist that she died of cholera asphyxia.

The "Cella," of the same line of steamers, arrived on the 20th from Havre with 360 passengers of the same class, and from the same region of country, but no case of sickness or death was reported during the passage and on arrival.

The "Mary Ann," an American bark, from Havre on the 25th of October, arrived on the 12th of December. The captain reported 5 deaths during the passage—4 from cholera; the first died on the 28th of October, and the three others on the 3d, 4th and 5th of November, after an illness of one to two days duration. On a small vessel, with a deck scarcely six feet high, and crowded to its utmost capacity, and without any special care or prevention, the disease disappeared, and all on board enjoyed good health for thirty days previous to her arrival in port.

The "Harpswell," which sailed on the 28th of October, a few days after the "Mary Ann," lost seven infants during the passage, but no cholera cases occurred. Equally exempt were the two first class steamers "Europe" and "America," with passengers directly from Paris, where the majority had resided for some time previous.

That cholera prevailed in Paris, and to some extent in Havre, has been admitted by all, and what is still more significant, the "Atlanta," "Mary Ann," "Hermann" and "Harpswell" had each names on the passenger list which were not among the passengers, but reported to have been sent to the hospital by the local authorities at Havre. The clean bills of health were unquestionably issued by the same spirit which reported 200 cases at Paris at a time when upwards of 300 daily died of cholera.

Although the appearance of cholera was not unanticipated in the port of New York, no facilities whatever were prepared for an efficient quarantine. The "Atlanta" was immediately, upon arrival, sent to the lower bay, the surgeon of the vessel relieved, and as soon as the hospital ship could be prepared and the weather admitted of the removal of the sick, they were all, as they occurred, transferred to the hospital ship; the baggage of the passengers was opened and aired; the soiled linen washed, and baggage, bedding, and personal effects of every kind subjected to fumigation in cool chambers prepared for that purpose. This fumigation was effected by a mixture of black oxide of manganese and common salt—equal parts well moistened—and the addition of sulphuric acid, one part to four. The generation of gas was so abundant that one of the hands of the boat could only be restored with difficulty after hours' attention, from the effects of inhaling the gas four hours after fumigation had commenced.

The quarantine of passengers has been decried as barbarous and inhuman; and certainly none would be more anxious to grant them better accommodations than the officer in charge. When we, however, consider that the disease is not in the vessel, but among her passengers, and will necessarily accompany them wherever they go, that the accommodations on board the vessel, if scanty, are at least adequate to their wants and such as they are accustomed to, the neglect of the authorities to provide proper accommodations, though not less fragrant, was at least shorn of its alleged inhumanity and barbarity; in fact, that debarcation does not eradicate the disease, any medical man will admit, and as an instance in proof I may mention the case of the "North America" in 1854. Cholera existed on board of that vessel two weeks before her arrival in port. Ten of her passengers had died during that time, and seven cases were sent to the hospital on her arrival. The day following all her passengers were landed. In three days 128 cases and 32 deaths occurred among 250 passengers, while the crew remained perfectly healthy, and no new cases could be traced to the vessel.

The passengers of the "Atlanta" received partique ten days after the occurrence of the last case, and the vessel, a few days afterwards, was thoroughly cleansed and repeatedly fumigated.

As facts are the only true basis of inference, I have limited my observations to simple recital of facts. Facts alone can guide us in a practical rational quarantine, and however much even medical men may differ as to the mode of its administration, all, I think, must agree upon the necessity of quarantine, both of sick and exposed.—*Medical and Surgical Reporter.*

